# Prediction of the Antioxidant Response Elements' Response of Compound by Deep Learning

**DOI:** 10.3389/fchem.2019.00385

**Published:** 2019-05-31

**Authors:** Fang Bai, Ding Hong, Yingying Lu, Huanxiang Liu, Cunlu Xu, Xiaojun Yao

**Affiliations:** ^1^School of Pharmacy, Lanzhou University, Lanzhou, China; ^2^School of Information Science and Engineering, Lanzhou University, Lanzhou, China; ^3^State Key Laboratory of Applied Organic Chemistry, Department of Chemistry, Lanzhou University, Lanzhou, China

**Keywords:** antioxidant response elements (AREs), deep learning, toxicity, prediction, machine learning

## Abstract

The antioxidant response elements (AREs) play a significant role in occurrence of oxidative stress and may cause multitudinous toxicity effects in the pathogenesis of a variety of diseases. Determining if one compound can activate AREs is crucial for the assessment of potential risk of compound. Here, a series of predictive models by applying multiple deep learning algorithms including deep neural networks (DNN), convolution neural networks (CNN), recurrent neural networks (RNN), and highway networks (HN) were constructed and validated based on Tox21 challenge dataset and applied to predict whether the compounds are the activators or inactivators of AREs. The built models were evaluated by various of statistical parameters, such as sensitivity, specificity, accuracy, Matthews correlation coefficient (MCC) and receiver operating characteristic (ROC) curve. The DNN prediction model based on fingerprint features has best prediction ability, with accuracy of 0.992, 0.914, and 0.917 for the training set, test set, and validation set, respectively. Consequently, these robust models can be adopted to predict the ARE response of molecules fast and accurately, which is of great significance for the evaluation of safety of compounds in the process of drug discovery and development.

## Introduction

Antioxidant response elements (AREs), a series of momentous regulators of redox homeostasis and activators of cytoprotection during oxidative stress, can be activated by the exogenous sources of oxidative stress to participate in a variety of diseases ranging from cancer to neurodegeneration diseases (Raghunath et al., 2018). AREs are crucial in a variety of physiological functions and interact with numerous transcription factors to arrange the expression of a batch of cytoprotective genes in a spatio-temporal manner (Ney et al., 1990). More specifically, AREs profoundly contribute to the pathogenesis and progression of carbohydrate metabolism, cognition, inflammation, iron metabolism, metastasis, reduced nicotinamide adenine dinucleotide phosphate (NADPH) regeneration, lipid metabolism, and tissue remodeling (Hayes and Dinkova-Kostova, 2014). As such, AREs are the vital targets of the signal transduction pathway in eukaryotic cells responded to oxidative stress and the prevention of potential chemical toxicity. Therefore, determining if one compound can activate AREs is crucial for the assessment of potential risk of compound.

Generally, the *in vitro* and *in vivo* evaluations of interactions between a large number of compounds and the AREs are expensive, time-consuming and labor intensive. Relatively, the *in silico* approaches can be used as an alternative way to predict if a compound can activate AREs with lower cost. Based on the advantages of *in silico* approaches, some machine learning-based methods have been proposed to predict the AREs activators in the environment (Huang et al., 2016). However, there are some problems to be solved in the development of prediction model, such as high false positive and low precision. Several model optimization strategies were also applied, such as bagging, consensus modeling, and feature selection (Drwal et al., 2015; Filip, 2015; Abdelaziz et al., 2016; Gergo, 2016; Yoshihiro, 2016). Although these strategies can be effective on some degree, the predictive performance of traditional machine learning-based methods still needs to be improved. Undoubtedly, the process of feature filtering avoids dimensional disasters, but results in the loss of relevant information. One of the most promising models for AREs' response prediction is the DeepTox developed by Mayr et al. (2016). Based on the Tox21 challenge data, they used deep neural network methods to predict AREs' response. The best model has the area under the Receiver Operating Characteristic (ROC) curve (ROC-AUC) with 0.840 and balanced accuracy with 0.677 on the validation set. Moreover, other models based on traditional machine learning methods, such as random forest (RF), support vector machine (SVM) and Naive Bayesian etc., displayed ROC-AUC ranging from 0.768 to 0.832 and the balanced accuracy ranging from 0.519 to 0.729 (Huang et al., 2016). From above all, the more reliable models for the prediction of AREs' response are still needed.

Recently, deep learning (Lecun et al., 2015), as a promising machine learning method, has been applied in a wide range of fields, such as physics, life science and medical science (Gulshan et al., 2016). There were also some researches in biology (Mamoshina et al., 2016; Dang et al., 2018; Hou et al., 2018) and drug design areas (Gawehn et al., 2016; Hughes and Swamidass, 2017). Furthermore, deep learning methods have been also applied in small molecule toxicity assessment (Blomme and Will, 2016). For example, deep neural networks (DNN) was applied to predict drug-induced liver injury (Xu et al., 2015; Fraser et al., 2018). Convolution neural networks (CNN) was applied to predict the acute oral toxicity (Xu et al., 2017). Relative to other machine learning methods, deep learning methods (Wu and Wei, 2018) have some special advantages. For example, deep learning does not require feature selection, which can make the maximum use of extracted molecular features. Secondly, deep learning integrates a multi-layered network that enables the integration and selective activation of molecular features to avoid overfitting problems. Thirdly, deep learning includes many different network structures and can analyze and classify the problems from different perspectives. All of these suggests that the emerging deep learning algorithms may help us build more reliable models to predict AREs' response of the studied compounds.

In this study, to build more reliable prediction model of AREs' response, a series of deep learning methods including deep neural networks (DNN), recurrent neural network (RNN), highway networks (HN), convolution neural networks (CNN) were applied on a large date set (Tox21 challenge data) including 8,630 compounds. For comparison, the traditional machine learning methods, random forest (RF) and support vector machine (SVM), were also applied to predict AREs' response.

## Materials and Methods

### Data Collection and Preparation

Tox21 challenge data^1^ (shown in Supporting Information) was used to build model. The structures of compounds was downloaded from PubChem^2^ according to the SID of compound. The AREs' response of compound was detected by CellSensor ARE-bla HepG2 cell line (Invitrogen), which was widely used to analyze the Nrf2/antioxidant response signaling pathway. To get the reliable data, each compound was tested in parallel by measuring the cell viability using CellTiter-Glo assay (Promega, Madison, WI) in the same wells. According to the test results, the molecules were categorized as “active,” “inactive,” or “inconclusion.” To keep the built models reliable, the molecules with label of “inconclusion” were removed. The three-dimensional conformations of molecules play a pivotal role in the development of prediction model (Foloppe and Chen, 2009). Therefore, all compounds used in this study were initially subjected to full geometry optimization in LigPrep (Schrödinger, 2015). During the geometry optimization, the energy minimization was carried out using OPLS2005 force field (Kaminski et al., 2001). The inorganic compounds, mixtures, counterions, tautomers, and the duplicates were removed to make sure each compound has only one optimized conformation. The ionizable groups were taken into consideration and the distinct conformations were produced with the pH window of 7.0 ± 0.2. In particular, the molecules were deleted if there were some unreasonable or improper structures. After these pretreatments, the remaining compounds include 1,136 active and 6,299 inactive compounds.

### Molecular Representation

The conventional molecular descriptors and molecular fingerprint features calculated by DRAGON 7.0 software (Kode srl, 2017) were used to describe the structural features of studied compounds. The calculated molecular descriptors include 0D (constitutional descriptors), 1D (functional groups counts, atom-centered fragments), 2D, and 3D-descriptors. The descriptors with missing values were removed. After this procedure, the number of remained molecular descriptors was 5,024. In general, the chemical features shared with those most active samples would be recognized to develop prediction models in the construction phase, while other chemical features shared with the least active molecules would be removed in order to avoid the complexity and increase the efficiency of models. The most relevant descriptors correlated with ARE toxicity were selected by Gini Index^3^.

Molecular fingerprints (FPs) encode the structural information of a molecule by exploding its structure in all the possible substructure patterns. By this method, a molecule is described as a binary string of substructure keys. Different substructure patterns with SMARTS lists are predefined in a dictionary, within which substructures are created as atom-centered fragments using a variant of Morgan's extended connectivity algorithm. For a SMARTS pattern, if a substructure was presented in the given molecule, the corresponding bit was set to “1” and otherwise set to “0.” In this study, the 1,024 bits extended connectivity fingerprints (ECFP) (Rogers and Hahn, 2010) were calculated by the DRAGON 7.0 program (Kode srl, 2017).

### Data Splitting

**Figure 1:**
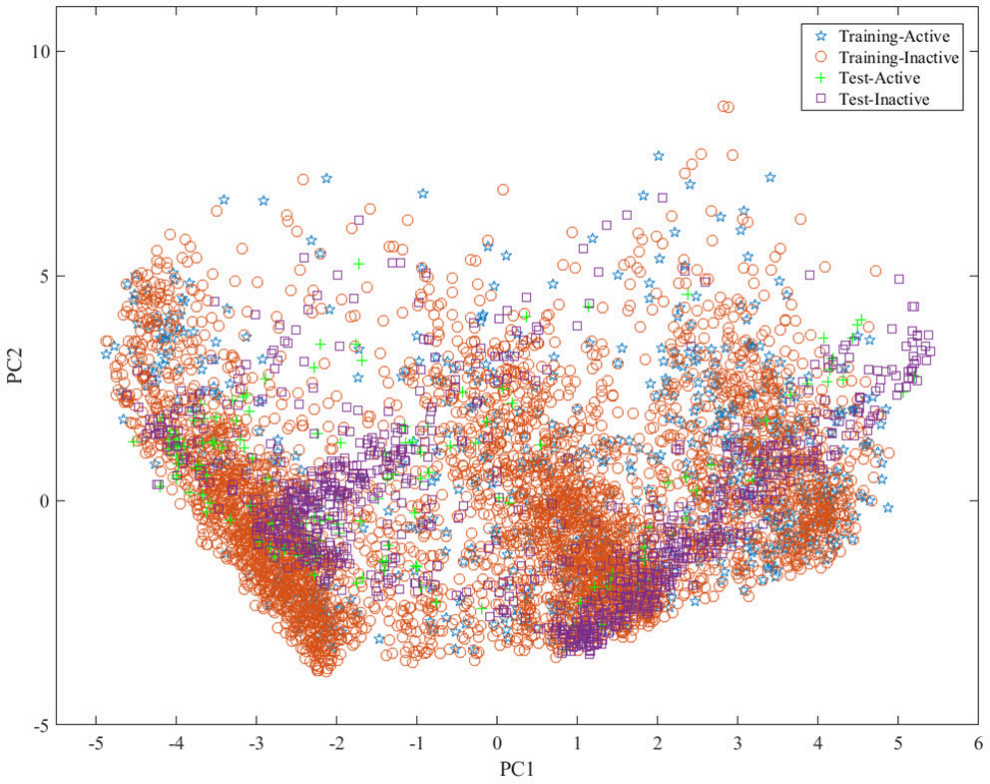
The distribution of samples in the training set and test set by principle component analysis (PCA) based on the molecular fingerprint features.

To build the reliable model, the representative data set should be selected to build and test model. For this aim, we divided the data set into training set, test set and validation set with the ratio of 4:1:1 by the Kennard and Stone algorithm (Kennard and Stone, 1969) by considering the structural features and activity of compound. The statistical summary of the data set was presented in Table 1. To show the distribution of compounds in training set and test set , principal component analysis (PCA)^4^ was performed based on the fingerprint features of compounds and the obtained results were shown in Figure 1, indicating that the compounds in training set and test set are well-distributed in the whole compound space.

**Table 1:** The statistical summary of the data sets.

	**Training set**	**Test set**	**External validation set**
Activation	756	190	190
Inactivation	4,199	1,050	1,050

### Machine Learning Methods

Recently, deep learning (Lecun et al., 2015) algorithms have been widely applied in a variety of areas and gave promising results (Mamoshina et al., 2016). Deep learning methods comprise a lot of architectures, such as deep neural networks (DNN), recurrent neural network (RNN), highway networks (HN), and convolution neural networks (CNN). The principle of the used deep learning methods was described as below. Due that the RF (Breiman, 2001) and SVM (Mavroforakis and Theodoridis, 2006) have been introduced elsewhere, here, their principle was not given again.

#### DNN Classifier

**Figure 2:**
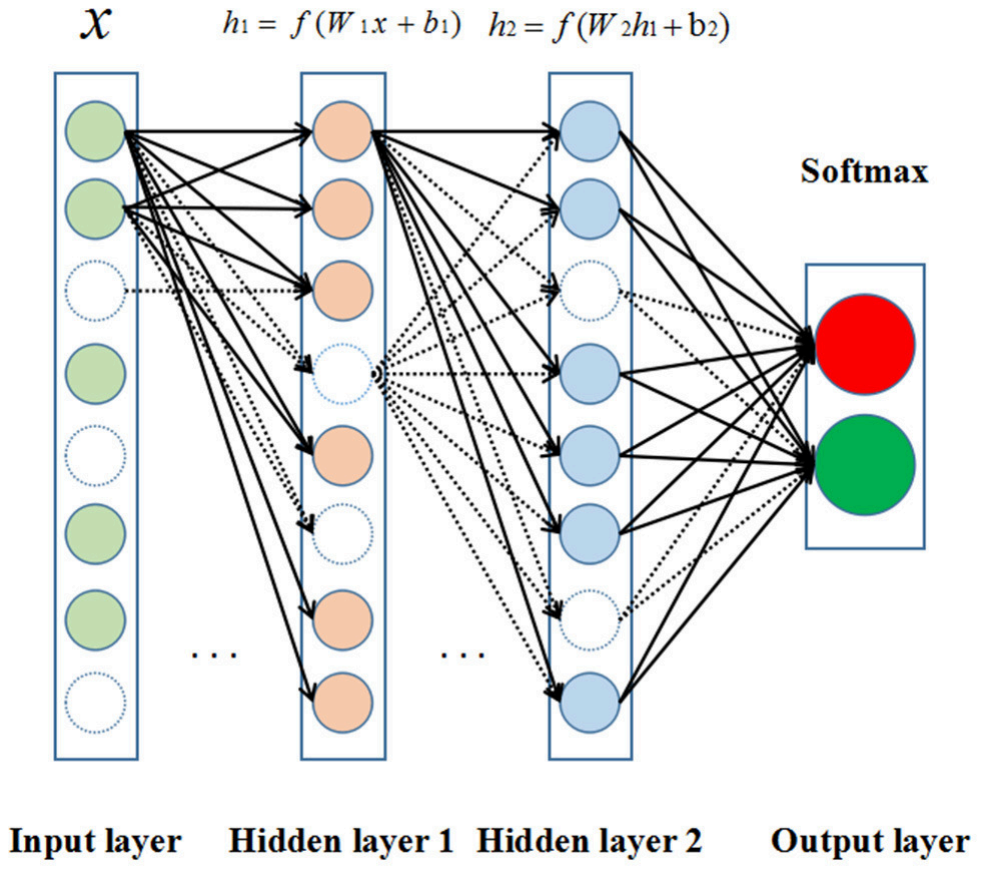
The structure of deep neural network (DNN). Neurons are represented by circles. The colored circles indicate the activated neurons while the circles without color are inactivated neurons. In addition, the arrows represent heavy-weight transmissions between neurons, and the dashed arrows mean the invalid neuronal connections.

The DNNs (Lecun et al., 2015) are developed from the structure of artificial neural networks with a large number of hidden layers. In the canonical deployment, the data are fed into the input layer and then transformed in a non-linear way through multiple hidden layers, and the final results are calculated and produced to the output layer. Neurons of hidden and output layer are connected to all neurons of the previous layer's. Each neuron calculates a weighted sum of its inputs and applies a non-linear activation function to generate its output as shown in Figure 2.

#### HN Classifier

The HNs (Srivastava et al., 2015) allows unimpeded information flow across several layers on information highways. The architecture is characterized by the use of gating units learning to regulate the flow of information through a network. HNs increases the possibility of studying extremely deep and efficient architectures for that it can be trained hundreds of layers directly with a variety of activation functions.

#### RNN Classifier

RNNs (Williams and Zipser, 1989) dedicates to process sequence data as it delivers state-of-the-art results in cursive handwriting and speech recognition. Its recent application in protein intrinsic disorder prediction demonstrated its significant ability to capture non-local interactions in protein sequences (Hanson et al., 2017). RNN processes an input sequence one element at a time, maintaining in its hidden units as a “state vector” that implicitly contains information about the history of all the past elements of the sequence. However, the training process becomes problematic for the backpropagated gradients either grow or shrink at each time step. After a batch of time steps they typically exploded or vanished (Hochreiter, 1991; Bengio et al., 2002). To solve the problem, a strategy was developed to augment the networks with an explicit memory-the long short-term memory (LSTM) networks. LSTM networks define special hidden units to remember the inputs for a long time (Hochreiter and Schmidhuber, 1997). A special unit called the memory cell acts like an accumulator or a gated leaky neuron. The cell has a connection to itself, so it copies its own real-valued state and it also accumulates the external signal at the same time. This self-connection mechanism decides whether to clear the content of the memory according to the other units states. LSTM networks have subsequently proved to be more effective than conventional RNNs, especially in several layers for each time step (Graves et al., 2013).

#### CNN Classifier

The CNNs (Krizhevsky et al., 2012) is a kind of multi-layer neural networks designed to process data fed in the form of multiple arrays. CNNs can exploit the property of many compositional hierarchies natural signals, owing to its ability of extracting higher-level features from lower-level ones. The architecture of typical CNN consists of three types of layers, which are convolutional, pooling, and fully-connected layers. Units in a convolutional layer are organized in feature maps. Each unit is connected to local patches of feature maps as well as previous layer through a set of weights called filter bank. After the process of convolutional layer, the new feature maps are obtained by applying a non-linear activation function, such as ReLU. The pooling layer is utilized to create an invariance filter to get small shifts and distortions by reducing the dimension of the feature maps. Each feature map of a pooling layer is connected to its preceding corresponding convolutional layers. The pooling layer computes the maximum of local patch of units in each feature map. And then the convolution and pooling layers are stacked by one or more fully-connected layers aiming to perform high-level reasoning feature generation (Hinton et al., 2012; Zeiler and Fergus, 2014).

### The Implementations of Machine Learning Methods

For deep learning methods, the MinMaxScaler was utilized to transform features, by which each feature was scaled into a given range between zero and one. The nodes in the network used both rectified linear units (ReLUs) and tanh functions as activation functions. The dropout algorithm (Hinton et al., 2012; Dahl et al., 2014) and L2 regularization were used to prevent overfitting. The model was trained using Adam (Adaptive Moment Estimation) optimizer (Tieleman and Hinton, 2012). Xaiver initialization was applied to initialize the parameters (Glorot and Bengio, 2010; He et al., 2015). Grid search method was employed to search the best hyperparameters. It should be noted that CNN model was built based on fingerprint features but not the descriptors, for the reason that CNN could only process highly correlated local regions of input sequences (Lecun et al., 2015). The other models were constructed based on both fingerprints and descriptors. All Deep Learning methods were implemented in Deep Learning framework of Tensorflow (version 1.5.0). All deep learning methods had 3 layers and with dropout rate of 0.1. The loss function was cross entropy. The other hyperparameters of the deep learning methods are listed in Table 2. The RF and SVM proceeded in Python scikit-learn (version 0.19.0) (Pedregosa et al., 2011). There were 80 trees in RF models. For SVM models, the kernel function was set as polynomial with gamma 0.1.

**Table 2:** The hyperparameters of deep learning methods.

**Models**	**Activation_function**	**Number of hidden units**	**Learning rate**	**Dropout rate**	**L2 weight decay**	**Epoches**
DNN	Tanh, relu, softmax	5,024, 32, 32	0.00001	0.1	0.01	30,000
HN	Tanh, relu, softmax	5,024, 32, 32	0.0001	0.1	0.01	3,000
RNN	Tanh, relu, softmax	5,024, 32, 32	0.0001	0.1	0.01	3,000
CNN	Relu, relu, softmax	Patch size 10^*^10	0.0001	0.1	none	2,000

### The Evaluation of Model Performance

The performance of generated models was evaluated by several statistic metrics, such as sensitivity (SE), specificity (SP), accuracy (ACC), Matthews correlation coefficient (MCC) (Fang et al., 2013), F_1_-score, and Precision. The formulas are shown as below:

$$\begin{array}{lll} SE & = & \frac{TP}{TP+FN}\\ SP & = & \frac{TN}{TN+FP}\\ ACC & = & \frac{TP+TN}{TP+TN+FP+FN}\\ MCC & = & \frac{TP\times TN-FN\times FP}{\sqrt{\left(TP+FN\right)\left(TP+FP\right)\left(TN+FN\right)\left(TN+FP\right)}}\\ F_{1} & = & \frac{2TP}{2TP+FP+FN}\\ Precision & = & \frac{TP}{TP+FP} \end{array}$$

Where TP, TN, FP, and FN refer to the numbers of true positives, true negatives, false positives, and false negatives, respectively. All these various validation requirements have been suggested to evaluate the model performance. The Receiver Operating Characteristic (ROC) curve and the area under ROC curve (ROC-AUC) were also calculated to evaluate the predictive ability of built model.

## Results and Discussion

### Performance Evaluation of Descriptors-Based Classification Models

**Figure 3:**
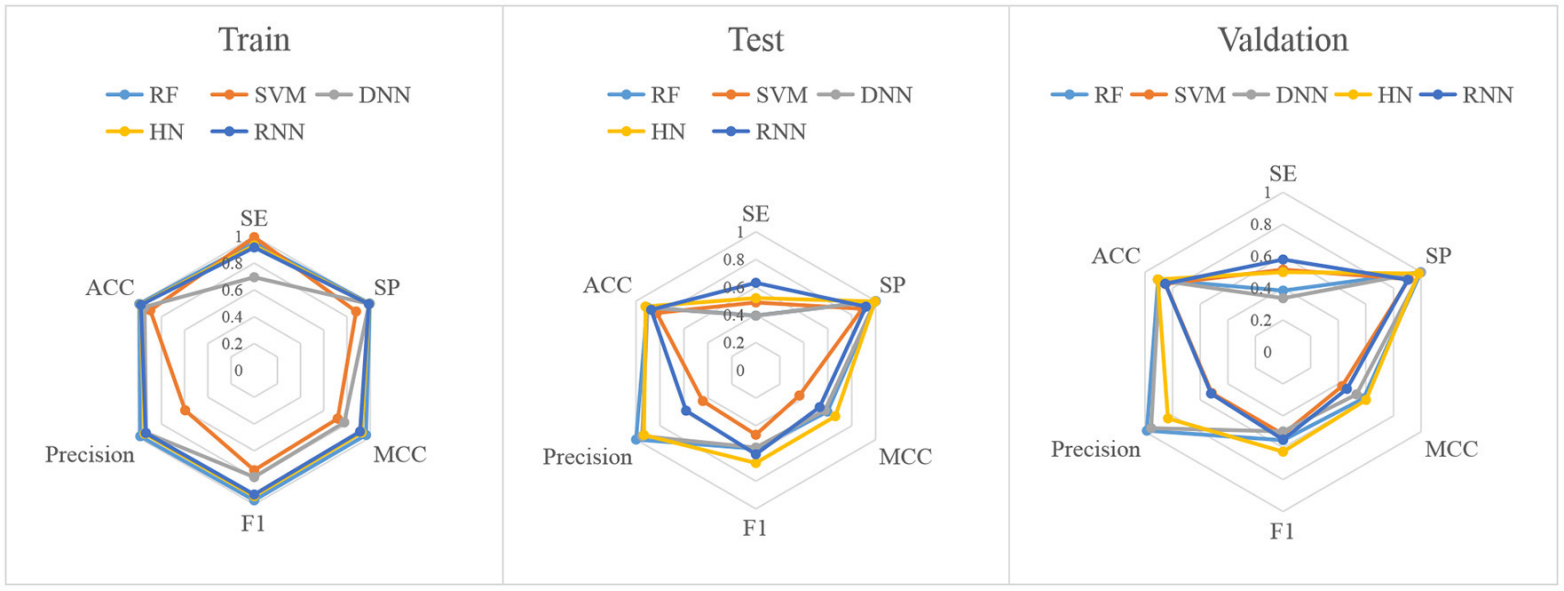
Radar plot of the descriptors-based classification models.

In this study, firstly, we employed various algorithms to build classification models based on molecular descriptors. The statistical evaluation of these models on the training set, test set and validation set are summarized in Table 3. For clarity, we have grouped all the metrics by training, test and validation sets and presented them as radar plots. A perfect score on all metrics would be represented by a circle the size of the complete plot. The shape of the plots can also be indicative of the quality of the models. The larger the circle is, the better the model is. The radar plots of ARE toxicity model based on the structural descriptors are shown in Figure 3.

**Table 3:** The performance of constructed models based on the general molecular descriptors.

**Methods**	**Group**	**TP**	**TN**	**FP**	**FN**	**SE**	**SP**	**MCC**	**F1**	**Precision**	**ACC**	**ROC_AUC**
RF	Tr	723	4,186	13	33	0.9563	0.9969	0.9638	0.9692	0.9823	0.9907	–
	Tst	75	1,050	0	115	0.3947	1.0000	0.5965	0.5660	1.0000	0.9073	0.8055
	Val	73	1,049	1	117	0.3842	0.9990	0.5828	0.5530	0.9865	0.9048	0.8298
SVM	Tr	751	3,689	510	5	0.9934	0.8785	0.7198	0.7447	0.5956	0.8961	–
	Tst	93	933	117	97	0.4895	0.8886	0.3631	0.4650	0.4429	0.8274	0.7755
	Val	98	958	92	92	0.5158	0.9124	0.4282	0.5158	0.5158	0.8516	0.7659
DNN	Tr	525	4,161	38	231	0.6944	0.9910	0.7766	0.7961	0.9325	0.9457	–
	Tst	75	1,046	4	115	0.3947	0.9962	0.5766	0.5576	0.9494	0.9040	0.8281
	Val	64	1,047	3	126	0.3368	0.9971	0.5321	0.4981	0.9552	0.8960	0.8573
HN	Tr	704	4,158	41	52	0.9312	0.9902	0.9270	0.9380	0.9450	0.9812	–
	Tst	99	1,043	7	91	0.5211	0.9933	0.6627	0.6689	0.9340	0.9210	0.7942
	Val	95	1,031	19	95	0.5000	0.9819	0.6008	0.6250	0.8333	0.9081	0.8267
RNN	Tr	693	4,151	48	63	0.9167	0.9886	0.9127	0.9259	0.9352	0.9776	–
	Tst	120	964	86	70	0.6316	0.9181	0.5320	0.6061	0.5825	0.8742	0.8287
	Val	110	949	101	80	0.5789	0.9038	0.4628	0.5486	0.5213	0.8540	0.8122

**Figure 4:**
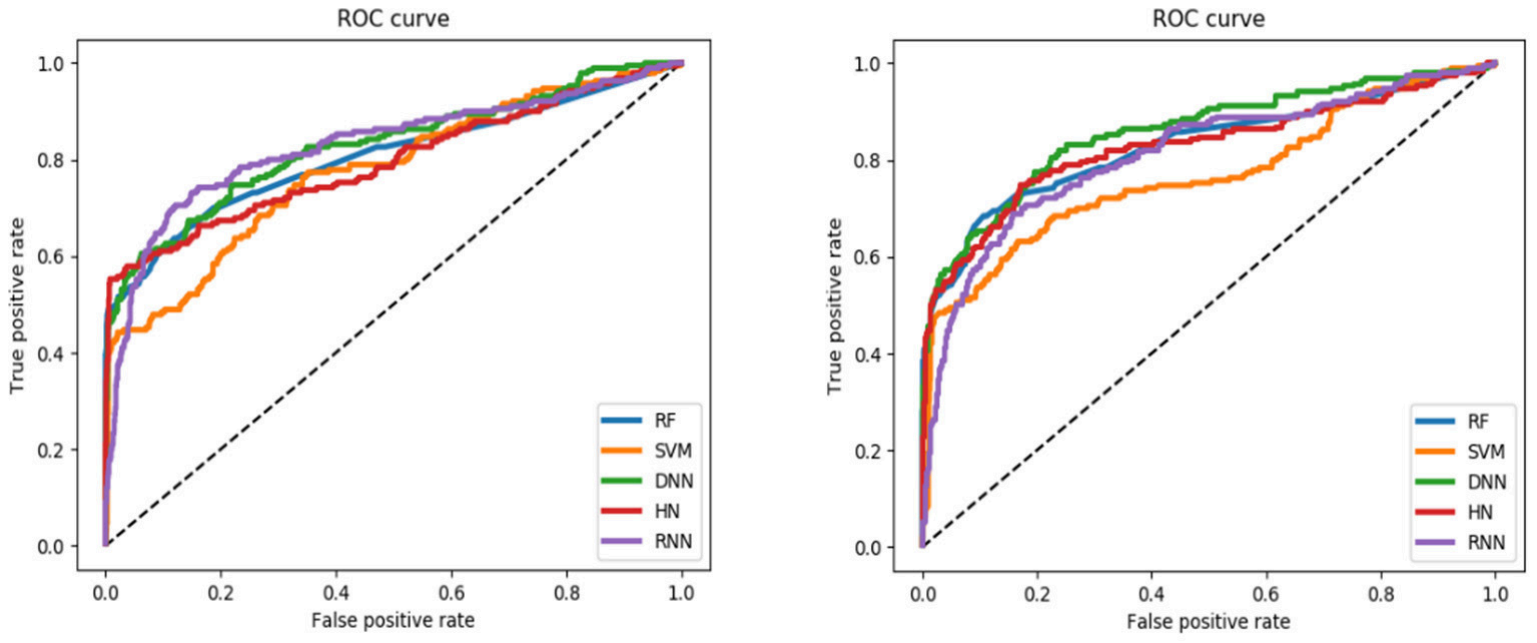
ROC curve of descriptors-based model (the left one is test set, the right one is validation set).

For the training set, all models gave very good SE, SP, MCC, F1-score, Precision, and ACC values. It should be noted that the SVM model showed lowest precision while DNN model exhibited lowest SE level. For the test and validation set, the indexes of all models exhibited a similar tendency, which tends to predict the compounds as inactivation due to the imbalanced distribution of active and inactive compounds. Among these models, the RNN model gave the highest SE value, while other indicators were not so well. It is worth noting that all indexes of the HN model were better than other models. In addition, the ROC-AUC is critical index for models performance and the ROC of all models are shown in Figure 4. For the test set, the RNN exhibited highest ROC-AUC (0.829), while for the validation set, DNN gave the highest ROC-AUC value of 0.857. Compared with the previous models, our models displayed a higher ROC value and ACC values.

In general, the DNN model performed well for the external validation set predictions from the ROC-AUC metric, while the HN exhibited the higher ACC (0.908) than DNN as well as the MCC and F_1_ with 0.601 and 0.625, respectively. The RF model gave higher SP (0.999) and Precision (0.986). On the contrary, the RNN method gave higher SE value (0.579) than other models.

We further analyzed what kinds of molecular properties will affect the ARE toxicity of compounds. The Gini index was applied to sort the importance of molecular descriptors. The top 20 descriptors and their corresponding meanings are shown in Table 4. From the information of selected descriptors, it is clearly that the walk and path count descriptors hold a great impact on the ARE toxicity of compound. The 3D matrix-based descriptors, the edge adjacency indices as well as the atom-type E-state indices are also significant for the ARE toxicity of compound. Besides, the 2D matrix-based descriptors and 2D autocorrelations P_VSA-like descriptors also have a close correlation with ARE toxicity of compound.

**Table 4:** 20 molecular descriptors selected by the RF method and Gini index analysis.

**Name**	**Meaning**	**Bolck**	**Sub-block**
TPC	Total path count	Walk and path counts	ID numbers
piPC09	Molecular multiple path count of order 9	Walk and path counts	Multiple path counts
PCR	Ratio of multiple path count over path count	Walk and path counts	ID numbers
ChiA_G	Average Randic-like index from geometrical matrix	3D matrix-based descriptors	Geometrical distance matrix (G)
Eig02_EA (bo)	Eigenvalue n. 2 from edge adjacency mat. weighted by bond order	Edge adjacency indices	Eigenvalues
StCH	Sum of tCH E-states	Atom-type E-state indices	E-State sums
piPC08	Molecular multiple path count of order 8	Walk and path counts	Multiple path counts
SM12_AEA (ri)	Spectral moment of order 12 from augmented edge adjacency mat. weighted by resonance integral	Edge adjacency indices	Spectral moments
SpDiam_B (m)	Spectral diameter from Burden matrix weighted by mass	2D matrix-based descriptors	Burden matrix weighted by mass (B (m))
SM13_AEA (ri)	Spectral moment of order 13 from augmented edge adjacency mat. weighted by resonance integral	Edge adjacency indices	Spectral moments
P_VSA_e_1	P_VSA-like on Sanderson electronegativity, bin 1	P_VSA-like descriptors	Sanderson electronegativity
GATS4s	Geary autocorrelation of lag 4 weighted by I-state	2D autocorrelations	Geary autocorrelations
SM02_AEA (bo)	Spectral moment of order 2 from augmented edge adjacency mat. weighted by bond order	Edge adjacency indices	Spectral moments
SM5_B (e)	Spectral moment of order 5 from Burden matrix weighted by Sanderson electronegativity	2D matrix-based descriptors	Burden matrix weighted by Sanderson electronegativity (B (e))
TDB01i	3D Topological distance based descriptors—lag 1 weighted by ionization potential	3D autocorrelations	TDB autocorrelations
Eta_betaS_A	Eta sigma average VEM coun	ETA indices	Basic descriptors
P_VSA_ppp_ar	P_VSA-like on potential pharmacophore points, ar—aromatic atoms	P_VSA-like descriptors	Potential Pharmacophore Points
SM5_B (i)	Spectral moment of order 5 from Burden matrix weighted by ionization potential	2D matrix-based descriptors	Burden matrix weighted by ionization potential (B (i))
SM4_B (v)	Spectral moment of order 4 from Burden matrix weighted by van der Waals volume	2D matrix-based descriptors	Burden matrix weighted by Van der Waals volume (B (v))
piPC02	Molecular multiple path count of order 2	Walk and path counts	Multiple path counts

### Performance Evaluation of Fingerprints-Based Classification Models

**Figure 5:**
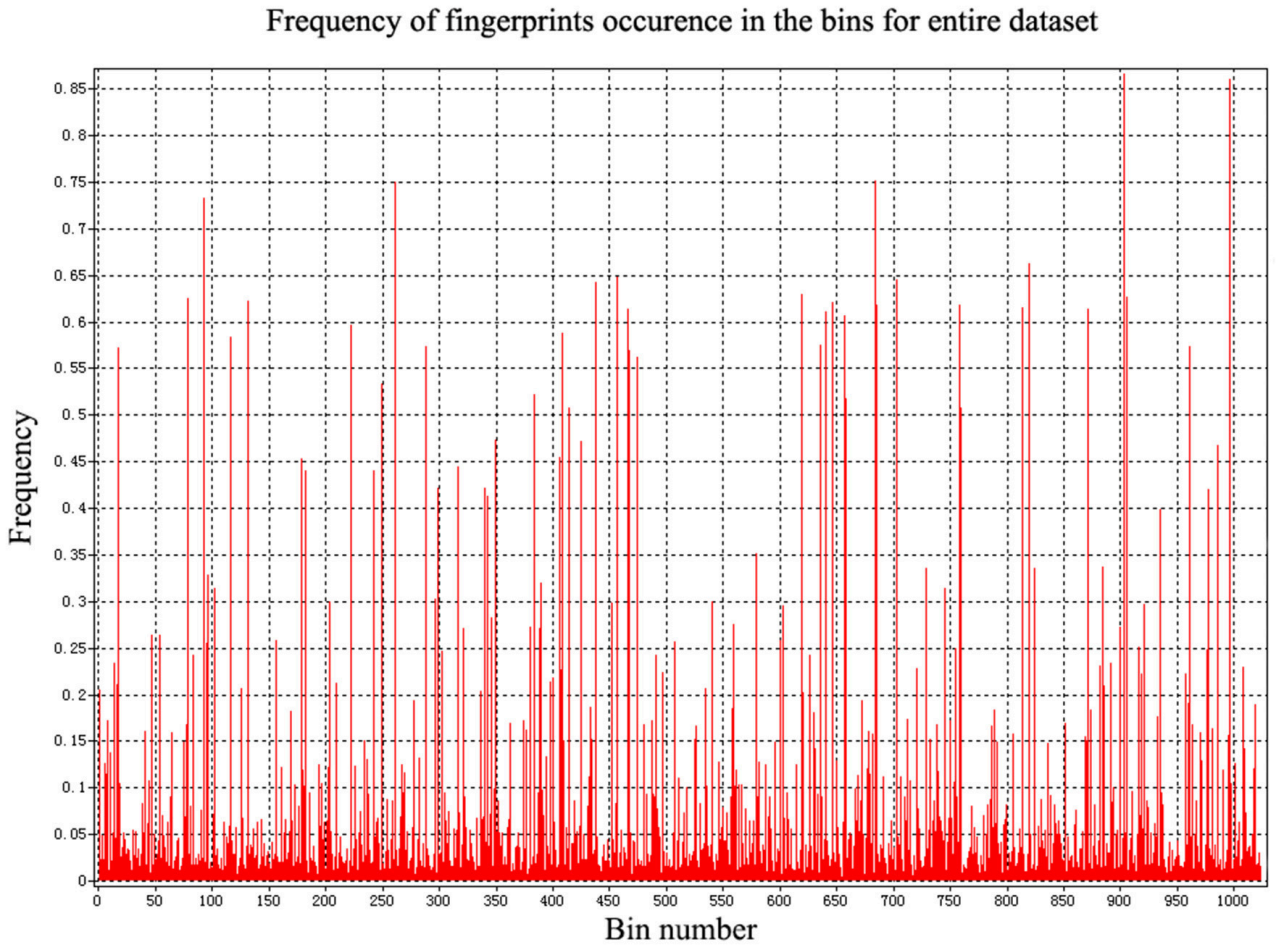
The frequency of fingerprints occurred in compounds.

**Figure 6:**
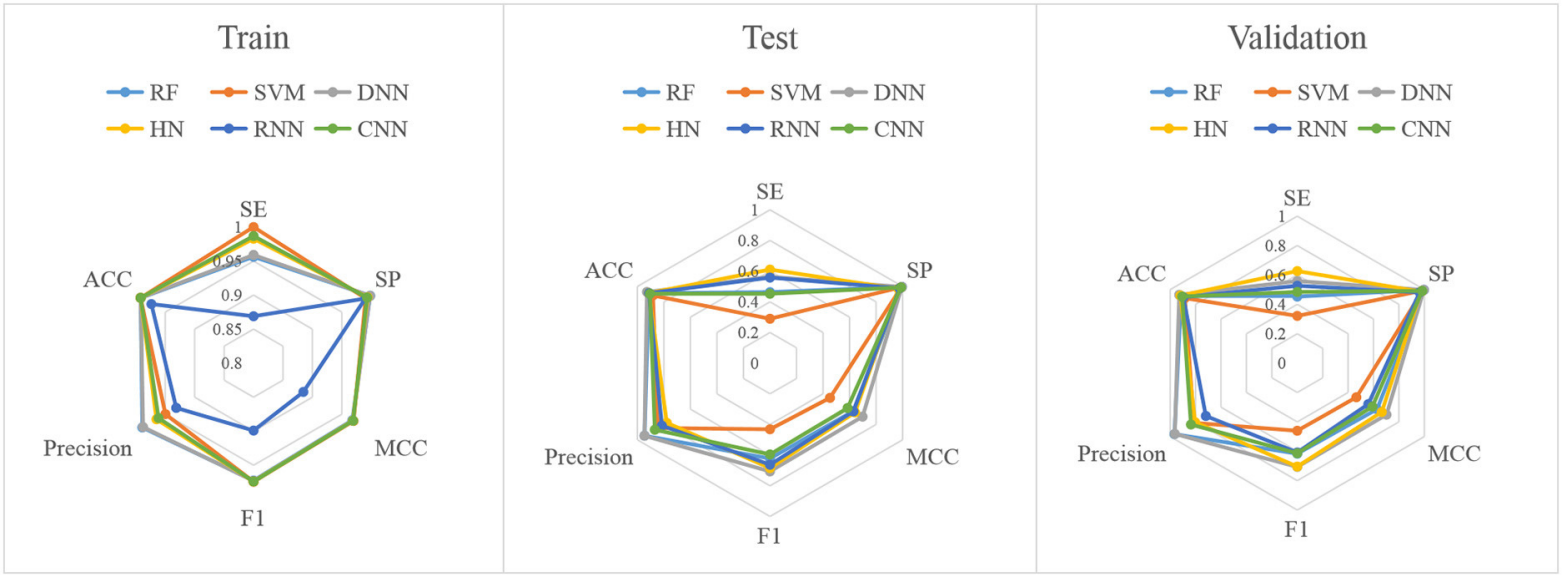
Radar plot of the fingerprints-based classification model.

In addition to the general molecular descriptors, the molecular fingerprint is another effective method to represent the structural features of molecules. A typical frequency of fingerprints occurred in the 1,024 bins of the compounds in the data set is shown in Figure 5. The fingerprints features were applied to build the six models including DNN, HN, RNN, CNN, RF, and SVM. The results are presented in Table 5 and the radar plots are presented in Figure 6.

**Table 5:** The performance of constructed models based on the fingerprints.

**Methods**	**Group**	**TP**	**TN**	**FP**	**FN**	**SE**	**SP**	**MCC**	**F1**	**Precision**	**ACC**	**ROC_AUC**
RF	Tr	723	4,191	8	33	0.9563	0.9981	0.9678	0.9724	0.9891	0.9917	–
	Tst	88	1,045	5	102	0.4632	0.9952	0.6269	0.6219	0.9462	0.9137	0.9613
	Val	86	1,047	3	104	0.4526	0.9971	0.6277	0.6165	0.9663	0.9137	0.9241
SVM	Tr	756	4,159	40	0	1.0000	0.9905	0.9699	0.9742	0.9497	0.9919	–
	Tst	55	1,040	10	135	0.2895	0.9905	0.4525	0.4314	0.8462	0.8831	0.8967
	Val	61	1,036	14	129	0.3211	0.9867	0.4650	0.4604	0.8133	0.8847	0.9049
DNN	Tr	725	4,190	9	31	0.9590	0.9979	0.9686	0.9732	0.9877	0.9919	–
	Tst	107	1,044	6	83	0.5632	0.9943	0.6977	0.7063	0.9469	0.9282	0.9607
	Val	106	1,046	4	84	0.5579	0.9962	0.7020	0.7067	0.9636	0.9290	0.9167
HN	Tr	743	4,172	27	13	0.9828	0.9936	0.9691	0.9738	0.9649	0.9919	–
	Tst	116	1,017	33	74	0.6105	0.9686	0.6415	0.6844	0.7785	0.9137	0.9329
	Val	119	1,021	29	71	0.6263	0.9724	0.6652	0.7041	0.8041	0.9194	0.8794
RNN	Tr	670	4,157	42	86	0.8862	0.9900	0.8982	0.9128	0.9410	0.9742	–
	Tst	106	1,026	24	84	0.5579	0.9771	0.6291	0.6625	0.8154	0.9129	0.9296
	Val	100	1,011	39	90	0.5263	0.9629	0.5585	0.6079	0.7194	0.8960	0.8534
CNN	Tr	746	4,169	30	10	0.9868	0.9929	0.9692	0.9739	0.9613	0.9919	–
	Tst	86	1,037	13	104	0.4526	0.9876	0.5851	0.5952	0.8687	0.9056	0.9329
	Val	92	1,032	18	98	0.4842	0.9829	0.5917	0.6133	0.8364	0.9065	0.8967

**Figure 7:**
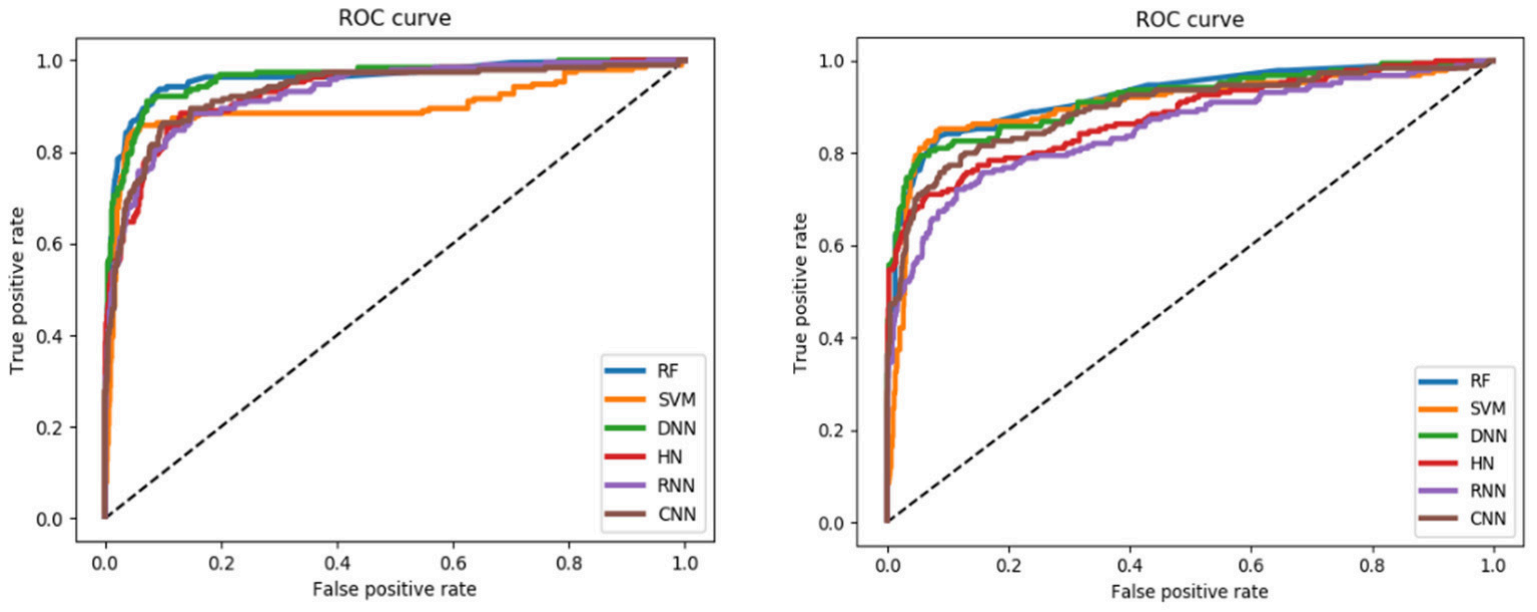
ROC curve of fingerprints-based model (**left:** test set, **right:** validation set).

For the training set, 5 out of all 6 models performed very well, except for the RNN method. According to the prediction results for test set, the value of SP, ACC, and precision were relatively stable, while the SE, F_1_-score and MCC showed different performance. The HN model exhibited the highest SE value while the SVM gave the lowest one. For the validation set, HN also performed better than other models on SE. As shown in Figure 7, all 6 models presented good ROC and large ROC-AUC, which were better than descriptor-based models. RF model has the highest ROC-AUC with 0.924 better than the DNN model with 0.917. However, the ACC of RF was lower than DNN model. But for the external validation set, Deep Learning methods had better generalization ability. Overall, the fingerprints-based models can give better prediction results than those based on molecular descriptors. The fingerprints of compounds were more useful than the descriptors for ARE toxicity prediction of compounds.

Compared with the traditional machine learning methods, deep learning methods had better learning ability and they could extract the inherent characteristics of the data. For the models based on the molecular descriptors, DNN showed highest ROC_AUC and ACC, while the HN exhibited the best SE performance. Considering the fingerprints features, the performance of DNN model was still well and HN showed higher SE than other models.

### The Comparisons Between Our Models and Other Models

We also compared the performance of our models with other reported models^5^. For the ARE toxicity prediction of Tox21 challenge data, the deep neural network models developed by Mayr et al. (2016) gave the best prediction results compared with other models. The best results they obtained had ROC-AUC 0.840, Balanced Accuracy 0.677 for the validation set. Other models displayed ROC-AUC ranging from 0.768 to 0.832 with the balanced accuracy between 0.519 and 0.729 using traditional machine learning methods, such as RF, SVM, and Naive Bayesian (shown in Table 6). Compared to their models and other models, our prediction models can give better prediction results. For the validation set, our best DNN model had ROC-AUC 0.917 and Accuracy 0.929.

**Table 6:** The reported top 10 prediction models of ARE toxicity prediction in Tox 21 challenge data set.

**Methods**	**ROC-AUC**	**Balanced accuracy**
DNN based on FP^a^	0.917	0.777
Bioinf@JKU	0.840	0.677
Bioinf@JKU-ensemble4	0.832	0.716
Bioinf@JKU-ensemble3	0.832	0.650
Bioinf@JKU-ensemble2	0.830	0.729
Bioinf@JKU-ensemble1	0.827	0.650
AMAZIZ	0.805	0.715
Microsomes	0.804	0.605
T	0.801	0.696
NCI	0.783	0.711
dmlab	0.768	0.519

a *FP means Fingerprints*.

## Conclusions

In this study, multiple deep learning algorithms were used to predict the ARE toxicity of compounds based on two kinds of molecular features including the general molecular descriptors and fingerprints. The DNN model based on fingerprints had an outstanding performance with ROC-AUC 0.917 and ACC 0.929, while the DNN model based on the general molecular descriptors had relative lower predictive ability with ROC-AUC 0.857 and ACC 0.896, suggesting that the fingerprints can represent the structural features of compounds related to their ARE toxicity more comprehensively. Compared with the traditional machine learning model, the deep learning models had much better predictive ability. Our constructed accurate predictive models on ARE toxicity will be valuable to the assessment of toxicity of compounds.

## Author Contributions

HL and CX conceived and designed the study. FB, DH, YL, and XY performed the experiment, analyzed the data, and wrote the manuscript.

### Conflict of Interest Statement

The authors declare that the research was conducted in the absence of any commercial or financial relationships that could be construed as a potential conflict of interest.

## Footnotes

^1^https://tripod.nih.gov/tox21/challenge/data.jsp

^2^https://pubchem.ncbi.nlm.nih.gov/

^3^https://en.wikipedia.org/wiki/Gini_coefficient

^4^https://en.wikipedia.org/wiki/Principal_component_analysis

^5^https://tripod.nih.gov/tox21/challenge/leaderboard.jsp

## Supplementary Material

The ID of compounds used in our model is available in the Supplementary Material.
