# Supplementary material for: Prediction of the Antioxidant Response Elements' Response of Compound by Deep Learning
Source: Front Chem. 2019 May 31;7:385. doi: 10.3389/fchem.2019.00385 (PMC6554289; doi:10.3389/fchem.2019.00385)
Supplement: Supplementary file 1 [file Table_1.DOCX]

**Supporting Information for:**

**Prediction of the antioxidant response elements’ toxicity by deep learning**

Fang Bai^§,†^, Ding Hong^§,‡^, Yingying Lu^#^, Huanxiang Liu^*,†^, Cunlu Xu^*,‡^, Xiaojun Yao^#^.

† School of Pharmacy, Lanzhou University, Lanzhou 730000, China

‡ School of Information Science & Engineering, Lanzhou University, Lanzhou 730000, China

# State Key Laboratory of Applied Organic Chemistry and Department of Chemistry, Lanzhou University, Lanzhou 730000, China

^§^ These two authors contributed equally.

*Corresponding Author:

Tel.: +86-931-891-2578

Fax: +86-931-891-2582.

E-mail: hxliu@lzu.edu.cn

E-mail: clxu@lzu.edu.cn

# Supplementary Data

The datasets for this study can be found in the National Institutes of Health (NIH) (https://tripod.nih.gov/tox21/challenge/about.jsp). The ID of active and inactive compounds are list in active-ID.csv and Inactive-ID.csv.
